# The past, present and future of opioid withdrawal assessment: a scoping review of scales and technologies

**DOI:** 10.1186/s12911-019-0834-8

**Published:** 2019-06-18

**Authors:** Joseph K. Nuamah, Farzan Sasangohar, Madhav Erranguntla, Ranjana K. Mehta

**Affiliations:** Industrial and Systems Engineering Department, Texas A and M University, College Station, TX 77843 USA

**Keywords:** Opioid use disorder, Surveys, Scales, Questionnaires, Wearable sensor, Physiological monitoring

## Abstract

**Background:**

A common challenge with all opioid use disorder treatment paths is withdrawal management. When withdrawal symptoms are not effectively monitored and managed, they lead to relapse which often leads to deadly overdose. A prerequisite for effective opioid withdrawal management is early identification and assessment of withdrawal symptoms.

**Objective:**

The objective of this research was to describe the type and content of opioid withdrawal monitoring methods, including surveys, scales and technology, to identify gaps in research and practice that could inform the design and development of novel withdrawal management technologies.

**Methods:**

A scoping review of literature was conducted. PubMed, EMBASE and Google Scholar were searched using a combination of search terms.

**Results:**

Withdrawal scales are the main method of assessing and quantifying opioid withdrawal intensity. The search yielded 18 different opioid withdrawal scales used within the last 80 years. While traditional opioid withdrawal scales for patient monitoring are commonly used, most scales rely heavily on patients’ self-report and frequent observations, and generally suffer from lack of consensus on the criteria used for evaluation, mode of administration, type of reporting (e.g., scales used), frequency of administration, and assessment window.

**Conclusions:**

It is timely to investigate how opioid withdrawal scales can be complemented or replaced with reliable monitoring technologies. Use of noninvasive wearable sensors to continuously monitor physiologic changes associated with opioid withdrawal represents a potential to extend monitoring outside clinical setting.

## Background

Opioids are natural (e.g., morphine), semi-synthetic (e.g., oxycodone) or synthetic (e.g., tramadol and methadone) narcotics primarily used to treat acute and chronic pain [1]. They are often used recreationally due to their euphoric, tranquilizing, and sedative qualities [2]. Misuse and abuse of opioids and associated overdose, referred to as opioid use disorder (OUD) [3], is a serious public health issue [4] and has been declared a public health emergency in United States [5]. Deaths due to opioid overdose continue to rise. For example, the number of opioid overdose deaths was 5 times higher in 2016 than in 1999, accounting for about 115 deaths per day [6].

OUD has significant societal impacts, such as escalating direct and indirect healthcare costs [7–9]. Florence et al. [8] estimated the total economic burden of prescription opioid overdose, abuse, and dependence for the year 2013 to be $78.5 billion. They found that more than a third of this amount ($28.9 billion) resulted from increased healthcare and substance abuse treatment costs. Indirect costs include lost workplace productivity [7], and criminal justice costs [7, 9]. In addition, the observed decline in men’s labor force participation from 1999 to 2015 has been associated with increased opioid prescriptions [10]. The opioid crisis is taking a toll on families, especially children, forcing an unprecedented number to enter foster care due to parental substance use [11].

Several treatment paths exist for OUD including opioid maintenance treatment, detoxification (detox) [2, 12], and medication-assisted treatment [13, 14]. Opioid maintenance treatment, a well-established first line approach to handle OUD [15], assesses suitability of opioid users who wish to start treatment, and provides legal substitutes. The detox process involves administration of medication in a controlled and medically supervised environment to reduce the severity of withdrawal symptoms that occur when people stop using opioids [16]. For opioid users who have expressed an informed choice, detox may be completed within a community-based program in up to 12 weeks or as inpatient in up to 28 days [16, 17]. Agonists, substances that fully bind to and stimulate opioid receptors (e.g., methadone, levomethadyl acetate) and partial agonists (e.g., Buprenorphine) medications are frequently used for both maintenance and detox purposes [12]. Medication-assisted treatment is an evidence-based treatment for OUD that combines behavioral therapy and approved medications including methadone, naltrexone, and buprenorphine [13, 14]. There is evidence suggesting that medication-assisted treatment is more effective than opioid maintenance treatment, and detox [13].

A common challenge with all OUD treatment paths is withdrawal management. Opioid users experience challenging and often severe withdrawal symptoms when they abruptly discontinue or reduce opioid intake. Intensity of opioid withdrawal symptoms depends on, among others, the type of opioid used, duration of usage, underlying medical conditions, and family history [18, 19]. Time frame for opioid withdrawal symptoms may be classified into early, peak, and late phases. The early phase is characterized by acute withdrawal symptoms such as lacrimation, yawning, and rhinorrhea. Symptoms including gastrointestinal symptoms, gooseflesh and aching reach their maximum intensity in the peak phase [19], and tail off in the late phase [18]. Detox that is not followed up with evidence-based treatment leads to a state of dysphoria which may expose patients to a high risk of relapse, and, even more tragically, a high risk of opioid overdose and death [20].

A prerequisite for effective opioid withdrawal management is early identification and assessment of withdrawal symptoms. One common assessment method for inpatient facilities (e.g., rehabilitation centers) is patient observation. This method involves frequent patient observations (in some cases every 1–2 h; [21]) and depending on the legal substitute used for treatment, clinicians may be required to take vital signs every 1.5 to 2 h with patient both sitting and standing [22]. Over the years, several withdrawal scales have been developed and used by clinicians to complement patient observation and to aid in assessing opioid withdrawal in outpatient cases. Most scales require patients to self-report their immediate or recent (e.g., in the past 24 h) symptoms experienced. Many signs and symptoms may be lost during the hours of the day when clinicians are not observing patients or when patients are not self-reporting. While efforts have aimed at improving the sensitivity and specificity of opioid withdrawal detection [23], the withdrawal scales have been associated with several limitations such as recall bias, distortion, and imprecision as well as the burden, both cognitive and psychological, due to recurring self-reporting (by patients) or observations (by caregivers) [24].

**Fig. 1:**
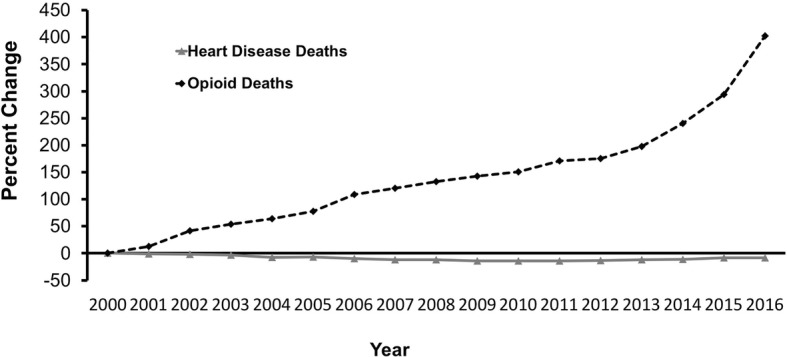
Percent change from baseline year 2000 of opioid deaths (8407 deaths in 2000) vs. heart disease deaths (728,796 deaths in 2000) from 2000 to 2016. For each category, percent change for each year was calculated as [(number of deaths in that year - number of deaths in year 2000)/number of deaths in year 2000] × 100. Data obtained from [6]

While opioid overdose deaths have increased tremendously over the past two decades such trend has been much more stable for chronic diseases, such as heart disease (Fig. 1). In fact, the downward trends in fatalities of other chronic diseases might be associated, in part, with advances in remote monitoring tools and techniques which may suggests that opioid withdrawal and relapse have lagged behind. For example, conditions such as diabetes, obesity, hypertension,

and chronic heart diseases have leveraged and benefited from advancements in smartphone and wearable sensor technologies (e.g., Jakicic et al. [25] monitored obesity using a multi-sensor device worn on the upper arm; Pedone, Chiurco, Scarlata, and Incalzi [26] monitored chronic obstructive pulmonary disease with a multi-sensor). In comparison, technologies to address and combat the opioid epidemic are few and are limited to diversion or monitoring overuse/misuse of prescription opioids (e.g., Electronic Prescribing for Controlled Substances, and CancelRx) with choice of services that would benefit patients [27]. Furthermore, a relatively small number of studies [28–30] have explored the use of biosensors to detect physiological changes associated with opioid intake in real-time.

Our initial steps to address this gap was to investigate available opioid withdrawal assessment methods to uncover opportunities for innovative technologies for withdrawal management. To the best of our knowledge, no study has critically reviewed and synthesized the commonly used opioid withdrawal scales. Additionally, there has not been any review of technological methods for opioid withdrawal management. The objective of this research was to review and document different opioid withdrawal monitoring methods, including surveys, scales and technology, to identify gaps in research and practice that could inform design and development of novel withdrawal management technologies.

## Methods

A scoping review of literature was conducted. We searched PubMed, Embase, and Google Scholar for articles published until April 19, 2019 using a combination of search terms (Table 1). We included studies if they (1) were written in English, (2) took place in an outpatient or inpatient setting, medication-assisted or opioid treatment program, rehabilitation center or were experiments (3) employed opioid withdrawal scale(s), wearable sensors, mHealth, or other technologies as predictor of opioid withdrawal severity (4) were peer-reviewed. In addition, we adopted the SPICE (setting, perspective, intervention, comparison, evaluation) framework, listed in Table 2. We excluded studies published in a language other than English, animal studies, alcohol or non-opiate studies, literature reviews, and non-peer-reviewed publications.

**Table 1:** Search terms used in the scoping review

Term	Search terms combination
Predictor	(“opi* withdrawal scale*” OR “biosensor*” OR “wearable sensor*” OR “opioid use disorder” OR “substance withdrawal syndrome” OR “opi* withdrawal syndrome” OR “withdrawal symptom*” OR “prescribed opioid use” OR “mHealth” OR “smart sensing tech*”)
Population	AND (“clinician*” OR “opioid patient*” OR “opioid addict*” OR “opioid user*” OR “caregiver*” OR “physician*” OR “doctor*” OR “surgeon*” OR “healthcare professional” OR “observer” OR “experimenter”)
Setting	AND (“inpatient” OR “outpatient” OR “opioid treatment cent*” OR “opioid treatment program*” OR “opiate treatment program*” OR “treatment cent*” OR “rehab* cent*” OR “medication-assisted treatment” OR “medication assisted treatment” OR “medication assisted treatment program*” OR “medication-assisted treatment program*” OR “medication-assisted treatment therapy” OR “medication assisted treatment therapy” OR “drug addiction cent”, OR “experiment”)
Evaluation	AND (“point score” OR “score” OR “severity of opi* withdrawal” OR “opi* withdrawal severity” OR “severity score” OR “severity of withdrawal” OR “withdrawal severity” OR “severity score” OR “score for severity” OR “withdrawal severity score” OR “withdrawal score for severity” OR “opi* withdrawal severity score” OR “opi* withdrawal score for severity”)
Exclusionary	NOT “animal studies” NOT “rat” NOT “mice” NOT “alcohol” NOT “non-opiate” NOT “literature review”

**Table 2:** SPICE criteria for inclusion of studies

Parameter	Inclusion criteria
Setting	Outpatient, inpatient, opioid treatment program, opioid treatment center, rehabilitation center, medication-assisted treatment program, medication-assisted treatment center, medication-assisted therapy, drug addiction centers, experiment
Population	Opioid patients, opioid users, opioid addicts, opioid dependents, caregivers, clinicians, doctors, surgeons, physicians, healthcare professionals, observer
Intervention	Opiate withdrawal scales, opioid withdrawal scales, biosensors, wearable sensors, monitoring, physiological monitoring, mHealth
Comparison	N/A
Evaluation	Determine severity of withdrawal

### Selection and triage

Article selection was carried out in two stages. In the first stage, two reviewers independently reviewed titles and abstracts against the inclusion and exclusion criteria using a web-based tool for systematic and scoping reviews called Rayyan [31]. The decision to fully review an article was made when both reviewers agreed to include the abstract. The reviewers resolved disagreements regarding article eligibility by discussing with a third reviewer.

**Fig. 2:**
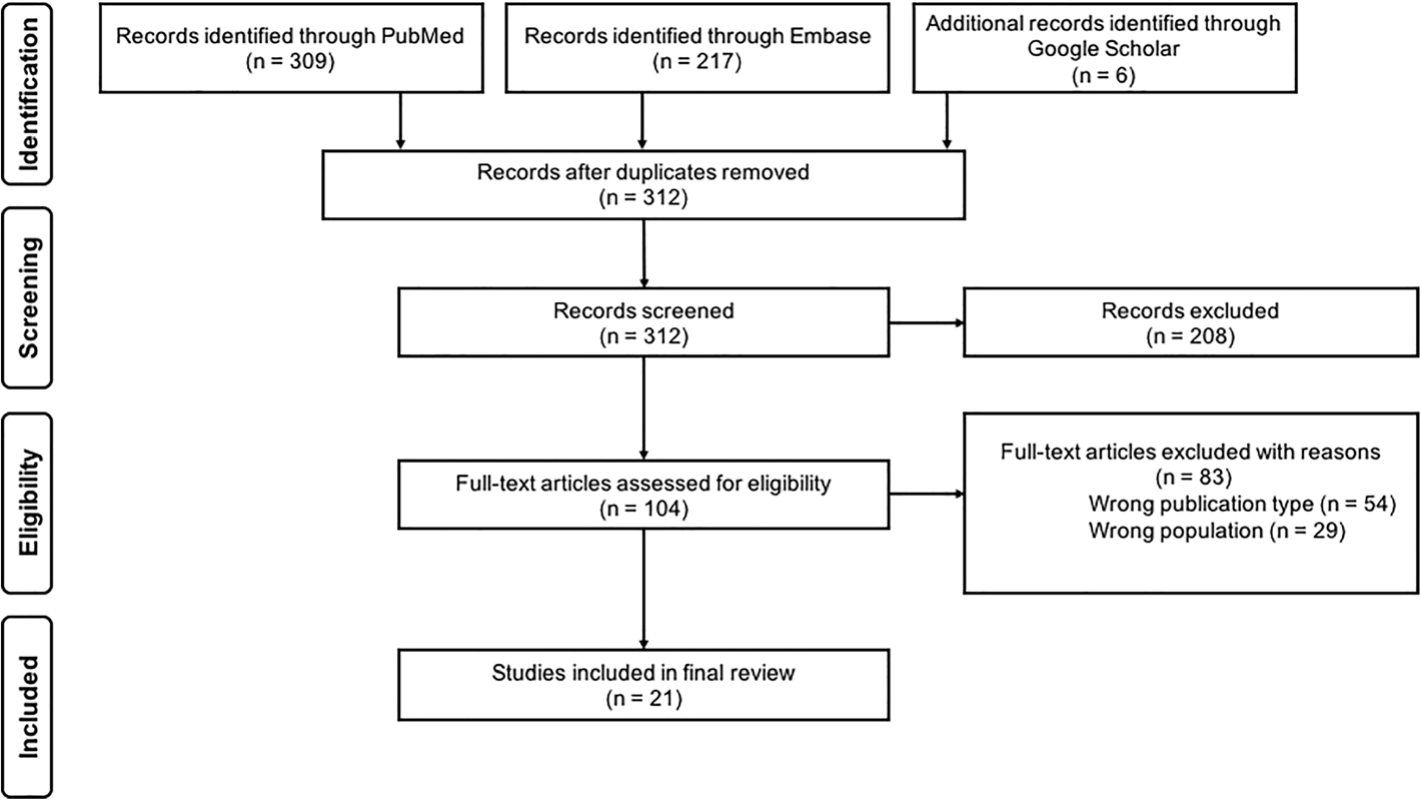
Process of searching and selecting articles included in the scoping review

In the second stage, the full-text articles were reviewed to determine eligibility. Furthermore, backward and forward reference search were conducted on all full-text articles that met the study selection criteria. Our initial search yielded 312 unique articles from which 104 articles were included after screening the titles and abstracts. Out of these 104 articles, 21 met the inclusion criteria and were included in the final review (see Fig. 2).

### Data extraction and analysis

Two reviewers independently read the full text of each article identified for inclusion in the review to extract pertinent data using a data extraction form. The SPICE framework was used to choose elements in the data extraction form. From each article, reviewers independently abstracted the following: setting (e.g., rehabilitation center), population (e.g., opioid user), intervention (e.g., scales, technology), and evaluation (e.g., severity of withdrawal). Reviewers transferred abstracted data to a detailed Excel spreadsheet. Authors met and organized the information extracted from articles into type of withdrawal assessment method, symptoms monitored, and temporal window covered. For the survey-based methods, authors also extracted information on scale name, mode of scale/survey administration and rating criteria employed. Date of scale development was inferred from when the study was first published.

## Results

### Type of assessments

Of the 21 articles that were included in the final review, 18 (86%) articles assessed opioid withdrawal using scales and surveys. Our search did not yield any empirical studies employing wearable sensors or mobile health (mHealth) apps to monitor opioid withdrawal symptoms. The remaining 3 (14%) articles employed technology to detect physiological changes during opioid intake.

### Scales and surveys to monitor opioid withdrawal

**Fig. 3:**
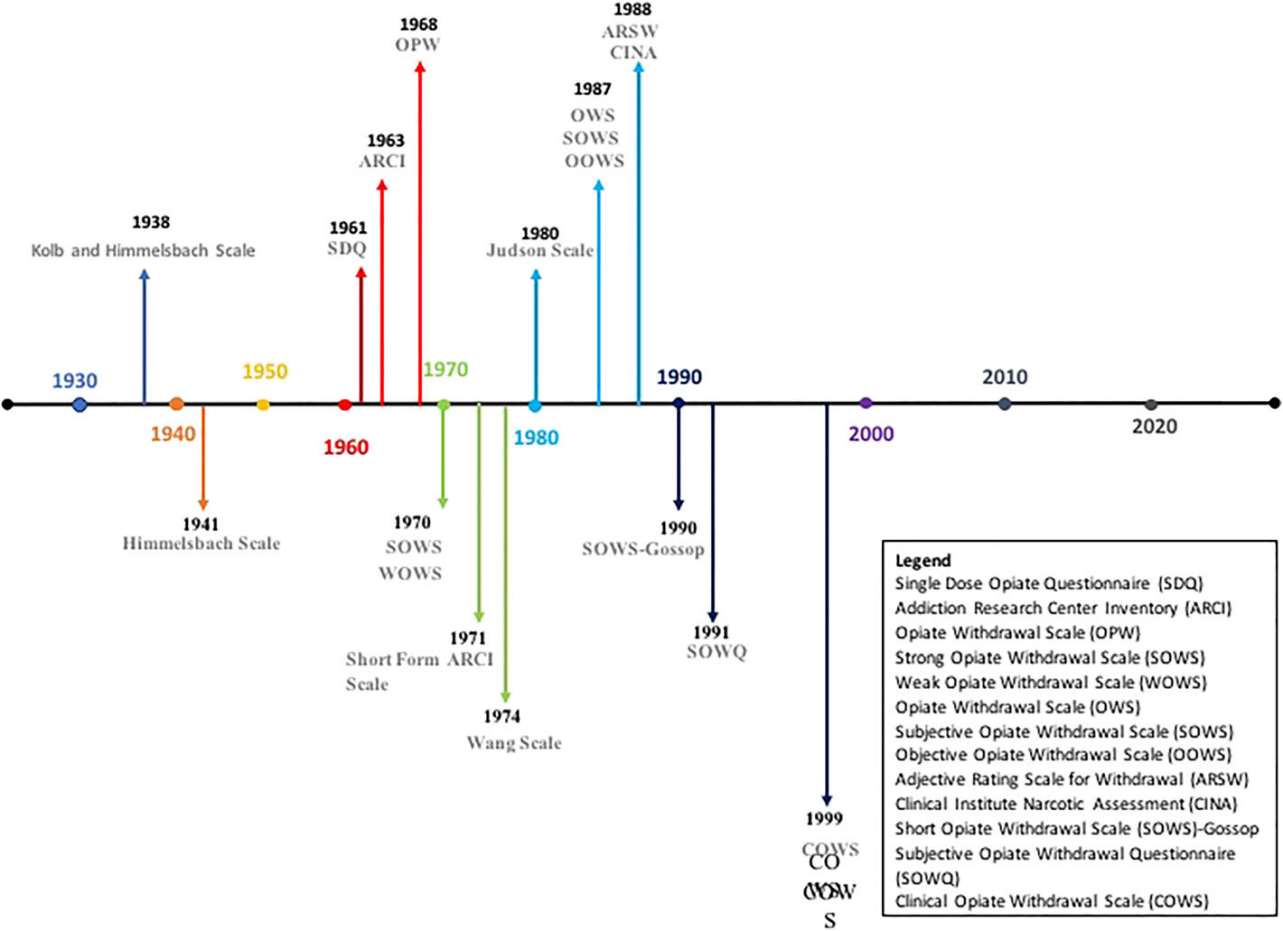
Timeline of opioid withdrawal scales development

The review revealed that opioid withdrawal scales are the main method of assessing and quantifying opioid withdrawal intensity. These scales employ a combination of observable behaviors (signs), patients’ self-reports (symptoms) and/or physiological measures. Overall, this search identified 18 different scales used over the past 80 years (see Fig. 3 for a visual timeline), dating back to Kolb and Himmelsbach’s [32] scale and the most recent being Clinical Opiate Withdrawal Scale (COWS) [19]. Table 3 provides a summary of the scales.

**Table 3:** Summary of opioid withdrawal scales (1938–2018)

Scale name	Mode of administration	Scale items	Rating criteria	Temporal window covered	Citation
Kolb and Himmelsbach Scale	Clinician-administered	14	Points-based	24 h	[33]
Himmelsbach Scale	Clinician-administered	14	Points-based	Hourly or 24 h	[34]
SDQ^1^ or Fraser Scale	Clinician-administered Patient-reported	6 (clinician) 4 (patient)	Weighted 5-point scale	15, 30, 45, 60, 90, 120 min post-drug	[45]
ARCI^2^	Patient-reported	550	True or False	Immediate feeling	[46]
OPW^3^	Patient-reported	29	5-point scale	Immediate feeling	[47]
WOW^4^	Clinician-administered	84	True/False	Immediate feeling	[35]
SOW^5^	Clinician-administered	80	True/False	Immediate feeling	[35]
Short Form ARCI	Patient-reported	49	True/False	Immediate feeling	[48]
Wang Scale	Clinician-administered	10	Points-based	10, 20, 30 min post injection	[37]
Judson Scale	Clinician-administered and Patient-reported	10 (clinician) 7 (patient)	4-point scale	Prior to zero time, and 10, 20, 30 min post injection	[38]
OWS^6^	Patient-reported	32	4-point scale	24 h	[39]
SOWS^7^	Patient-reported	16	5-point scale	Immediate feeling	[40]
OOWS^8^	Clinician-administered	13	Present/Absent	10-min observation period	[40]
ARSW^9^	Patient-reported	16	10-point scale	24 h	[42]
CINA^10^	Clinician-administered	13	Point-based	5, 10, 15 mins post injection	[49]
SOWS-Gossop	Patient-reported	10	4 -point scale	24 h	[43]
SOWQ^11^	Patient-reported	20	Anchored 100 mm analogue scale	24 h	[44]
COWS^12^	Clinician-administered	11	Weighted scale	2-min observation period	[19]

^1^Single Dose Opiate Questionnaire

^2^Addiction Research Center Inventory

^3^Opiate Withdrawal Subjective Experience Scale

^4^Weak Opiate Withdrawal Scale

^5^Strong Opiate Withdrawal Scale

^6^Opiate Withdrawal Scale

^7^Subjective Opiate Withdrawal Scale

^8^Objective Opiate Withdrawal Scale

^9^Adjective Rating Scale for Withdrawal

^10^Clinical Institute Narcotic Assessment

^11^Subjective Opiate Withdrawal Questionnaire

^12^Clinical Opiate Withdrawal Scale

Most of the scales used today are modeled after the Himmelsbach’s [33] scale, which assesses withdrawal syndrome intensity based on changes in observable behaviors (yawning, lacrimation, rhinorrhea, perspiration, tremor, restlessness, emesis, gooseflesh) and physiological measures (hyperpnea, systolic blood pressure, rectal temperature, weight). Using this scale, the total opioid withdrawal syndrome intensity score is computed as the sum of the points scored by each item on the scale.

The scales developed in the 1960s – Single Dose Opiate Questionnaire (SDQ), Addiction Research Center Inventory (ARCI), Opiate Withdrawal Subjective Experience Scale (OPW), focused on subjective effects of opioid withdrawal. To further study these effects, Haertzen, Meketon, and Hooks [34] developed two clinician-administered questionnaires – the 84-item Weak Opiate Withdrawal Scale (WOW) and the 80-item Strong Opiate Withdrawal Scale (SOW) to measure less intense and more intense withdrawal respectively.

With the emergence of methadone maintenance programs in the 1970s, physicians had a harder time establishing diagnosis of opiate dependence. Some patients mastered the art of going from one methadone program to the next, mimicking symptomatology of acute withdrawal to get a large initial dose of methadone. This widespread behavior caused a need for an objective scale to determine late-stage acute opiate withdrawal and withdrawal severity [35]. Researchers (e.g., [36]) developed protocols to precipitate withdrawal in order to assess the level of physical dependence on opioids. Using these protocols, the Wang scale [37], Judson scale [36], Opiate Withdrawal Scale (OWS) [38], Subjective Opiate Withdrawal Scale (SOWS) [39], and Objective Opiate Withdrawal Scale (OOWS) [39] sought to control feigned responses and improve on sensitivity and specificity for detecting withdrawal.

A number of patient-reported outcome instruments have been developed to measure acute symptoms of opioid withdrawal symptoms. These instruments developed to measure withdrawal include OWS [38], SOWS [39], Adjective Rating Scale for Withdrawal (ARSW) [40], Short Opiate Withdrawal Scale (SOWS)-Gossop [41, 42], and Subjective Opiate Withdrawal Questionnaire (SOWQ) [43].

SOWS-Gossop, Clinical Institute Narcotic Assessment (CINA) and Clinical Opiate Withdrawal Scale (COWS) are the most widely used instruments to evaluate opiate withdrawal symptoms in the reviewed articles [19, 22]. SOWS-Gossop [42] is a 10-item, patient-reported scale on which each item is rated as 0 (none), 1 (mild), 2 (moderate), or 3 (severe). It is a short version of the 32-item OWS [38]. CINA is a validated 13-item clinician-administered scale that measures opioid withdrawal signs and symptoms [44]. COWS [19] is a widely used 11-item clinician-administered instrument mostly due to its time-efficiency (i.e., can be completed within 2 min).

#### Mode of administration

Nine out of 18 available scales are clinician-administered only, 8 are patient-reported only, and 2 are both clinician-administered and patient-reported. Clinician-administered questionnaires are completed using traditional paper and pencil while clinicians talk face-to-face to patients and observe for opioid withdrawal signs. Patient self-administered questionnaires (patient-reported) require patients to complete questionnaires by hand and return them to the clinician. Scales that use both clinician-administered and patient-reported questionnaires (e.g., SDQ) concurrently but independently require both patients and clinicians to use traditional paper and pencil to complete questionnaires. None of the reviewed literature reported on which mode of administration is preferred or provides utility.

#### Number of scale items

The scales vary in the number of scale items (i.e., signs and/or symptoms) included, ranging from as many as 550 scale items (e.g., ARCI) to as few as 10 items (e.g., SOWS-Gossop). For example, the 550 items on the ARCI subjectively measure drug-induced effects as well as effects associated with personality and psychiatric disorders. The short-form of the ARCI has 49 items that allows for quick administration, and repeated measurements. On the other hand, OWS [38] is a 32-item patient-reported checklist of opiate withdrawal symptoms, with SOWS-Gossop [42] being a 10-item winnowed version.

**Fig. 4:**
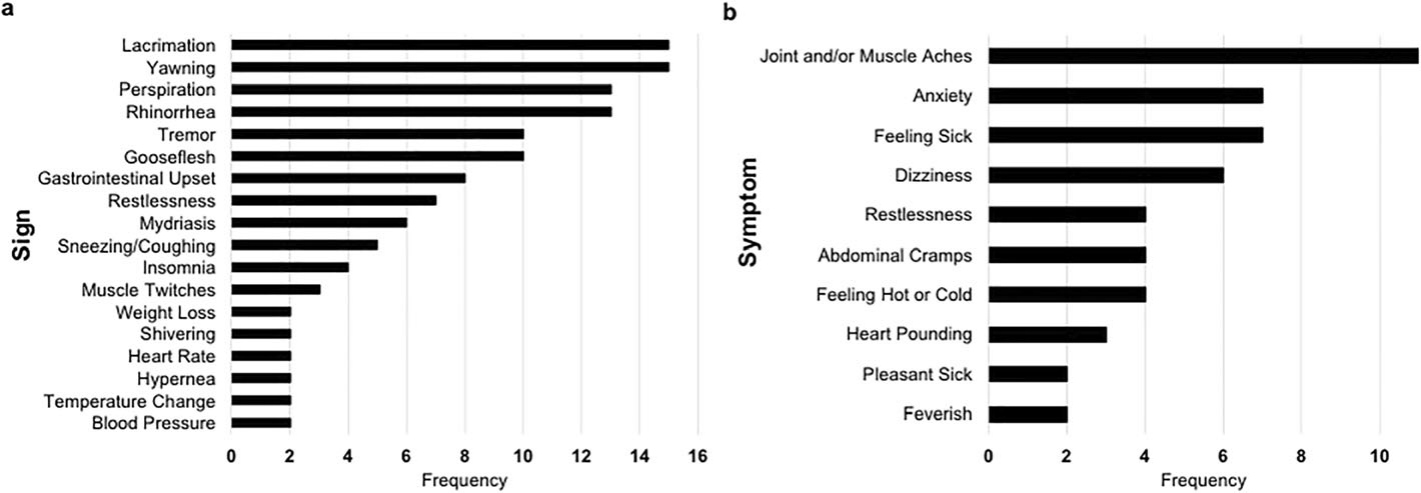
**a** Signs versus number of scales they appeared on; **b** Symptoms versus number of scales they appeared on

Scale items are made up of signs and/or symptoms. Whereas a symptom is a manifestation of a disease apparent to a patient alone, a sign is a manifestation of a disease that a clinician and/or patient can perceive [48]. Thus, while signs are usually observed by the clinician, symptoms are self-reported by patients. Lacrimation and yawning were the most frequent signs, appearing on 15 out of the 18 scales. Weight loss, shivering, heart rate, hypernea, temperature change and blood pressure were the least frequent signs, appearing on 2 out of 18 scales (see Fig. 4a). Joint and/or muscle aches was the most frequent symptom, appearing on 11 out of 18 scales (see Fig. 4b). The least frequent scales were pleasant sick and feverish. One of the most widely used scales, COWS, assesses both signs and symptoms. Items include anxiety or irritability, gastrointestinal upset, restlessness, bone or joint aches, sweating, rhinorrhea, tremor, gooseflesh, yawning, pupil size, and pulse rate.

#### Rating criteria

The scales differ in their use of rating criteria. For each item on a scale, various response categories are used to rate its clinical severity. Across all scales, ratings for items are summed up to create a total score, with a higher score indicating greater severity. Five (ARCI, WOW, SOW, Short Form ARCI, OOWS) of the scales used binary rating systems (“true/false” or “present/absent”). Three out of these 5 scales are clinician administered and the remaining 2 are patient-reported. Here, the total score for each scale is the sum of “true” or “present” items. Only 1 scale, SOWQ, employed a 20-item anchored analog scale. Patients rate each item on a 100 mm analogue scale anchored on both ends by pairs of pleasant and unpleasant feelings. The total score is the sum of analog scores for all 20 items [43].

While most scales use Likert scale ratings, there is no agreement on the type of scale with four (Judson Scale, OWS, SOWS-Gossop out of 18 scales use a 4-point rating, 2 (OPW, SOW) out of 18 use a 5-point rating, 3 (Kolb and Himmelsbach Scale, Himmelsbach Scale, Wang Scale) out of 18 use point-based rating, and 1 (ARSW) out of 18 scales uses a 10-point rating system. One scale, SDQ, uses a weighted 5-point rating system, for which physiological measures are given highest weights than behavioral signs, followed by patient reported symptoms. Many of the studies did not provide the rationale behind choice of points or weights for items on the scales.

The scales also differ in the value of total score. For example, whereas scores for the 11-item CINA range from 0 to 31 and higher scores are associated with more severe withdrawal syndrome, scores for the 11-item COWS range from 0 to 47 with specific ranges for the level of severity (e.g., scores from 5 to 12 are considered mild, scores from 13 to 24 are considered moderate, scores from 25 to 26 are considered moderately severe and scores more than 36 are considered severe withdrawal).

#### Temporal window covered

Temporal window covered differ among the scales depending, in part, on the nature of the legal substitute used (e.g., methadone or naloxone) to precipitate withdrawal, mode of drug administration (e.g., sublingual or intramuscular), and mode of scale administration. Five out of 18 scales, mostly patient-administered, have 24-h post-drug temporal window. Six scales require addicts to report their “immediate feeling”. The remaining scales varied markedly in temporal window covered, ranging from a 2-min observation period for COWS to 10, 20, 30 min post injection for the Wang scale.

### Technologies to monitor opioid intake

Three studies employed wearable biosensors to detect physiological changes during opioid intake. In all three studies, the biosensors were attached to the wrist as a band and the common biomarkers that were monitored included electrodermal activity (EDA), skin temperature, and whole-body acceleration. The temporal windows covered were before, during, and after opioid administration.

The first of these studies, a preliminary observational one, employed a wristband biosensor (Q sensor™, Affectiva, Waltham, MA) to continuously measure electrodermal activity (EDA), skin temperature, and physical movements of 4 patients before, during, and after cocaine and opioid administration. They found that patients with extensive opioid use demonstrated little to no change in EDA while low-moderate users of opioid showed rise in EDA and decrease in skin temperature [28]. In a follow up study, Carreiro et al. [29] employed the same wristband biosensor to continuously measure EDA, skin temperature, and physical movements of 30 patients before, during, and after naloxone administration. They found that although EDA did not show significant difference post administration compared to baseline, participants’ average skin temperature was significantly higher after naloxone administration. Overall movement was found to have decreased significantly after drug administration [29]. In the most recent study, Mahmud et al. [30] collected EDA, skin temperature and accelerometer data from 30 participants who wore the same wristband to develop an automated real-time system that detects opioid intake with 99% accuracy.

## Discussion

The scoping review of different opioid withdrawal monitoring methods revealed greater research efforts and emphasis on scales/surveys to monitor symptoms compared to technologies, which focused solely on symptoms associated with opioid uptake. While limited, advancements in non-invasive and wearable sensor technology could potentially serve as efficient complementary solutions in managing withdrawal symptoms either in inpatient or remote settings.

### Challenges with existing monitoring methods

Our results show that opioid withdrawal scales are and continue to be the main instrument used to assess and quantify opioid withdrawal symptoms. Over the years, scales have been developed either to assess the degree of physical dependence (before buprenorphine or methadone induction) and/or to compare the efficacy of opioid withdrawal treatments [19]. However, it is very difficult to compare these scales because of their wide heterogeneity, especially, with respect to the number of scale items, rating criteria, and temporal window covered.

CINA, COWS, and SOWS-Gossop appear to be the most widely used in research and clinical settings [19, 22]. In particular, COWS, designed to be administered by a clinician, is known to be more practical and relatively easy to use [19, 22]. However, with respect to the other scales there is no evidence suggesting the usability, practicality or lack thereof. In addition, while there are overlap and similarities between the scales, evidence of cross-validation is largely absent. Future work is needed to investigate context-dependent factors contributing to adoption and efficacy of different scales in different settings.

Monitoring of opioid withdrawal with scales can be conceptualized as sampling of patients’ behavior or experience over time. However, not all signs and symptoms may be captured by questionnaires or retrospective reports of behavior [50]. Reliance on global assessments or retrospective reports prevents clinicians from accurately understanding and characterizing dynamic behavioral changes over time and across situations in both real-world and clinical settings [51].

Another issue is that opioid withdrawal scales that require subjective patients’ reports ask for recall or summary information over long periods of time. Patients’ contexts and mental states at the time of recall often bias their memory retrieval process [50]. This exposes these scales to recall bias [51] which may affect the quality and reliability of the data provided. Indeed, opioid withdrawal scales may be considered as patient-reported outcome measures (PROMs) [52]. A patient-reported outcome is any information on the outcome of healthcare directly reported by a patient without interpretation or modification by a clinician [53]. Administering PROMs that measure subjective symptoms, like opioid withdrawal scales, requires consideration of issues about data collection – data source (self vs. proxy), mode of administration (self vs. interviewer) and method of administration (paper-and-pencil vs. electronic), and scoring [54]. These issues have limitations which complicate scoring and analysis of opioid withdrawal scales response data [54]. Scales that require direct response may be limited by patients’ cognitive or communication abilities. Those that require a proxy to respond about a patient’s experiences may not accurately represent the patient’s subjective experiences [54]. Scales that require patients to self-administer and record responses have potential for missing data, whereas those that require an interviewer to read questions and record responses suffer from interviewer costs and interviewer bias [55]. Scales that are administered using paper-and-pencil are time consuming and are prone to data entry errors [56]. Patient self-administered PROMs using technology minimizes data entry errors, can provide immediate scoring and are amenable to incorporation within electronic health records [57]. Scores for most PROMs, including opioid withdrawal scales, are based on the classical test theory, and are computed as a linear combination of all items on a particular PROM [58, 59]. For a score on an opioid withdrawal scale to be considered valid, all items must be used, making it test-dependent [54, 58, 59].

The findings suggest that some opioid withdrawal scales may impose high length, complexity and frequency of administration to users. Lengthy questionnaires have been identified as a general obstacle in clinical practice [60] and may lead to increased respondent burden and response fatigue, leading to reduced completion, and reduced data quality [61]. While more recent scales are relatively short, they still require physical and cognitive efforts to understand the questions, recall information from memory, evaluate the connection between the retrieved information and the question, and communicate their responses [62]. Furthermore, patient-reported scales demand that patients do not have visual impairments and are able to read and write in the language of the questionnaire. Since some scales require several observations and measurements per day, there is a need to support clinical decision-making by facilitating the comparison between measurements (e.g., to determine trends over time). No evidence was found in the reviewed literature suggesting that this need is currently fulfilled.

### Potential technological solutions

The current method of monitoring opioid withdrawal using scales is challenging outside of clinic or research environments. An opioid monitoring method that accurately monitors withdrawal symptoms as they occur in real time would provide several distinct advantages including the ability to obtain environmental and behavioral contexts surrounding withdrawal symptoms [28], additional source of information does not rely on self-reports and does not suffer from recall bias, and the potential for advanced data analytics, trend analysis, and decision support.

Using noninvasive wearable sensors, including temperature, accelerometer, electrodermal activity, and photo plethysmography sensors, to continuously monitor physiologic changes associated with opioid withdrawal represents a potential to extend monitoring outside clinical setting [63]. Sensors have shown promise to measure symptoms such as tremor, joint/muscle aches [64], gooseflesh [65], and anxiety [66]. Unlike opioid withdrawal scales, wearable technologies have the advantage of using biosensors to continuously measure and record physiologic changes in multiple contexts [67]. Continuous biosensing may provide a way to decrease opioid withdrawal observation time, and possibly allow for remote monitoring [68].

Even though remote monitoring technologies have shown promise in the management of chronic diseases (e.g., [60, 61]), application of such technologies in opioid withdrawal monitoring has been very limited. Short- and long-term physiological symptoms associated with opiate withdrawal timelines have been established [18]. This implies that wearable smart sensing technologies may be used to effectively and reliably identify, evaluate, and communicate physiological responses associated with opioid withdrawal and may inform clinical decision support tools and self-management technologies, with minimal burden to patients or care providers. Additionally, by retroactively examining patient-specific data on withdrawal symptoms and future doses, information can be provided back to clinical researchers to derive better intervention strategies.

While sensor-enabled remote monitoring tools to assess signs and symptoms of opioid withdrawal shows potential, several important challenges remain to be investigated. Most importantly, patient engagement and compliance have consistently shown to be problematic in other domains utilizing such tools and technologies [69, 70], particularly diabetes [71]. Work is needed to understand if such tools and technologies will be used on a sustainable manner and if not, investigate contextual factors contributing to such behaviors.

### Limitations

There are some limitations in the study that warrant discussion. The scoping review utilized relatively fewer, albeit relevant, number of databases to identify potentially eligible studies. Despite this limitation, we found saturation in the types and descriptions of subjective methods for opioid withdrawal monitoring (e.g., surveys and scales). The limited findings pertaining to technology-based methods may likely be due to the lagging nature of technology development for opioid withdrawal and/or uptake monitoring. It is also possible that technology design processes are not effectively captured and disseminated using traditional peer-reviewed analysis approach. Indeed, to capture emerging trends in technologies for opioid withdrawal monitoring, non-empirical investigations like technology landscape analysis of commercial technologies (e.g., mHealth apps) may be more appropriate [72].

## Conclusions

While traditional opioid withdrawal scales for patient monitoring are commonly used, most scales rely heavily on patients’ self-report and frequent observations, and generally suffer from lack of consensus on the criteria used for evaluation, mode of administration, type of reporting (e.g., scales used), frequency of administration, and assessment window. Therefore, it is timely to investigate how such scales can be complemented or replaced with reliable monitoring technologies. Smart sensing technologies that provide real-time information on the physiological and psychological status of patients have shown promise in addressing a similar need for other conditions such as diabetes, cardiovascular diseases, and mental health and may significantly, and proactively, improve treatment care of opioid patients by keeping clinicians aware of patient status.

Work is in progress to identify viable technologies to assess a wide range of withdrawal symptoms more objectively. This includes a comprehensive systematic review and analysis of past, present and future trends in wearable sensor technologies. Our long-term goal is to develop a technological framework using a human-centered design process that is non-invasive, reliable, and proactive to detect and manage opiate withdrawal symptoms by utilizing an array of sensors that can be employed to detect temporal and spectral patterns of physiological and psychological responses associated with short- and long-term withdrawal symptoms.

## Data Availability

Not applicable.

## Abbreviations

ARCI: Addiction research center inventory
ARSW: Adjective rating scale for withdrawal
CINA: Clinical institute narcotic assessment
COWS: Clinical opiate withdrawal scale
Detox: Detoxification
EDA: Electrodermal activity
OOWS: Objective opiate withdrawal scale
OPW: Opiate withdrawal subjective experience scale
OUD: Opioid use disorder
OWS: Opiate withdrawal scale
PROMs: Patient-reported outcome measures
SDQ: Single dose opiate questionnaire
SOW: Strong opiate withdrawal scale
SOWQ: Subjective opiate withdrawal questionnaire
SOWS: Subjective opiate withdrawal scale
SOWS-Gossop: Short opiate withdrawal scale-Gossop
SPICE: Setting, perspective, intervention, comparison, evaluation
WOW: Weak opiate withdrawal scale
