# Correction to: The past, present and future of opioid withdrawal assessment: a scoping review of scales and technologies

**DOI:** 10.1186/s12911-019-0851-7

**Published:** 2019-07-08

**Authors:** Joseph K. Nuamah, Farzan Sasangohar, Madhav Erraguntla, Ranjana K. Mehta

**Affiliations:** Industrial and Systems Engineering Department, Texas A and M University, College Station, TX 77843 USA

**Correction to: BMC Med Inform Decis Mak**

**https://doi.org/10.1186/s12911-019-0834-8**

Following publication of the original article [1], the authors reported an error in one of the authors’ names. In this Correction the incorrect and correct author name are shown. The original publication of this article has been corrected.

Originally the author name was published as:

Madhav Erranguntla

The correct author name is:

Madhav Erraguntla
